# Two colonic endoscopic submucosal dissections with a single multipolar adjustable traction device: an ecological tip to reduce waste and cost

**DOI:** 10.1055/a-2410-3135

**Published:** 2024-09-25

**Authors:** Elena De Cristofaro, Jérôme Rivory, Tanguy Fenouil, Louis-Jean Masgnaux, Jean Grimaldi, Pierre Lafeuille, Mathieu Pioche

**Affiliations:** 1 60259 Gastroenterology, University of Rome Tor Vergata Faculty of Medicine and Surgery, Rome, Italy; 2 36609 Gastroenterology and Endoscopy Unit, Hôpital Edouard Herriot, Lyon, France; 3 36609 Institute of Pathology, Hôpital Edouard Herriot, Lyon, France

Endoscopic resection is recommended by current guidelines for visible colitis-associated neoplasia in patients with ulcerative colitis (UC) 1 . Endoscopic submucosal dissection (ESD) is advised in selected cases to ensure en bloc resection, thereby reducing the risk of recurrence and need for colectomy during follow-up; however, its widespread use is limited by the prolonged time it requires and its technical difficulty 2 .

To address these challenges, various techniques have been described to facilitate and speed up the procedure, including traction strategies 3 4 . We have previously described the use of an adaptive traction device (ATRACT) to facilitate the resection of a giant dysplastic colonic lesion in a UC patient 5 . Herein, we detail the application and benefits of the ATRACT-2 device to resect two adjacent flat lesions located in the sigmoid colon of a 69-year-old patient with UC ( Video 1 ).

A single adaptative traction device is used to resect two adjacent colonic lesions in a patient with ulcerative colitis.Video 1

After circumferential incision and trimming had been performed for the first (distal) lesion, the two loops of ATRACT-2 were fixed by clips to the edges of the lesion. The rubber band was affixed to the opposite wall and the dissection was commenced with traction assistance. The traction device was tightened after half of the lesion had been cut to restore proper traction for completion of the dissection. The same traction device was subsequently used for the second (proximal) lesion, with one of the loops fixed to the edge of the lesion after it had been incised and trimmed, ensuring adequate submucosal exposure ( Fig. 1 ). Both procedures were successfully completed in a total of 35 minutes, without any adverse events. Histopathological analysis revealed two sessile serrated lesions without dysplasia.

**Fig. 1:**
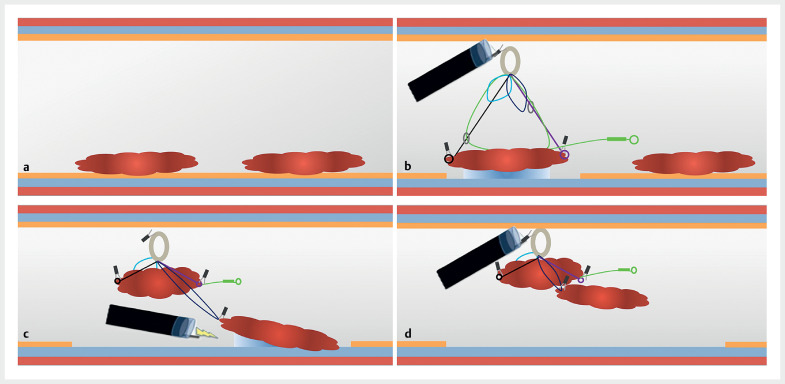
Schematic representation of the adaptive traction strategy used for two adjacent colonic lesions showing: **a** two adjacent colonic lesions; **b** traction applied to the distal lesions using the ATRACT-2 device; **c** traction applied to the proximal lesion using the same device; **d** retrieval of the two lesions simultaneously.

We can infer that this strategy can ensure good submucosal exposure in adjacent lesions using a single traction device, allowing for a reduction in the total number of clips. In addition, the lesions can be retrieved together without using additional devices. This could be considered an ecological tip to reduce waste and costs during procedures on adjacent lesions.

Endoscopy_UCTN_Code_TTT_1AQ_2AD_3AD

## Notes

Endoscopy E-Videos https://eref.thieme.de/e-videos
*E-Videos* is an open access online section of the journal *Endoscopy* , reporting on interesting cases and new techniques in gastroenterological endoscopy. All papers include a high-quality video and are published with a Creative Commons CC-BY license. Endoscopy E-Videos qualify for HINARI discounts and waivers and eligibility is automatically checked during the submission process. We grant 100% waivers to articles whose corresponding authors are based in Group A countries and 50% waivers to those who are based in Group B countries as classified by Research4Life (see: https://www.research4life.org/access/eligibility/ ). This section has its own submission website at https://mc.manuscriptcentral.com/e-videos .
